# Challenging cases of professionalism in Japan: improvement in understanding of professional behaviors among Japanese residents between 2005 and 2013

**DOI:** 10.1186/s12909-015-0313-6

**Published:** 2015-03-11

**Authors:** Kensuke Kinoshita, Yusuke Tsugawa, Peter B Barnett, Yasuharu Tokuda

**Affiliations:** 1 Department of Medicine, Mito Kyodo General Hospital, University of Tsukuba, Mito City, Ibaraki Japan; 2 Harvard Interfaculty Initiative in Health Policy, Cambridge, MA USA; 3 Office of Medical Education, University of Nevada School of Medicine, 1664N Virginia St, Reno, NV 89557 USA; 4 Japan Community Healthcare Organization, Tokyo, Japan

## Abstract

**Background:**

Professionalism is deemed as the basis of physicians’ contract with society in Japan. Our study in 2005, using a questionnaire with scenarios to professionalism, suggested that many physicians at various levels of training in Japan encounter challenges when responding to these common scenarios related to professionalism. It is unclear how medical professionalism has changed among Japanese residents in over time.

**Methods:**

We conducted a follow-up survey about challenges to professionalism for Japanese residents using the same Barry Questionnaire after a seven-year interval from the prior survey. The survey uses six clinical scenarios with multiple choice responses. The six cases include the following challenges: acceptance of gifts; conflict of interest; confidentiality; physician impairment; sexual harassment; and honesty in documentation. Each scenario is followed by 4 or 5 possible responses, including the “best” and the “second best” responses. The survey was conducted as a part of nationwide general medicine in-training examination.

**Results:**

We collected data from 1,049 participants (290 women, 28%; 431 PGY-1 and 618 PGY-2 residents). Overall, the current residents performed better than their colleagues in the earlier survey for five scenarios (gifts, conflict of interest, confidentiality, impairment, and honesty) but not for the harassment scenario. PGY-2 residents were more likely to select either the best or 2nd best choices to gifts (p = 0.002) and harassment (p = 0.031) scenarios than PGY-1 residents. Residents in the current study chose either the best or 2nd best choices to the gifts (p < 0.001) and honesty (p < 0.001) scenarios than those of the previous study conducted seven years ago, but not for the harassment scenario (p = 0.004).

**Conclusions:**

Our study suggests that there is improvement of medical professionalism with respect to some ethical challenges among the Japanese residents in the current study compared to those in our previous study.

**Electronic supplementary material:**

The online version of this article (doi:10.1186/s12909-015-0313-6) contains supplementary material, which is available to authorized users.

## Background

Medical professionalism is defined as the ability to meet the ethical expectations required to practice medicine competently [1]. These expectations are based on the principles of patient welfare, autonomy and social justice. Professionalism should be manifested through a commitment to carrying out professional responsibilities, adherence to ethical principles, and sensitivity to a diverse patient population [2]. In Japan, professionalism is seen as the basis of physicians’ contract with society, and public trust depends on the integrity of both individual professionals and the profession as an entity [3]. Sociocultural context is likely to have influence on the conceptual framework and practice of medical professionalism in different countries. Japanese physicians are more likely to set professional altruism as a moral priority based on the bushido spirit from the Edo era [3-5].

There are several responsibilities among physicians, including competence, honesty, confidentiality, appropriate relations with patients, improving quality of care, improving access to care, just distribution of finite resources, commitment to scientific knowledge, maintaining trust by managing conflicts of interest, and commitment to professional responsibilities [2,6]. As such, professionalism is viewed as one of the five core competencies as defined by ACGME, and is expected to be part of a structured curriculum and assessment process in both undergraduate and post-graduate medical education. On the other hand, it is challenging to formally educate medical professionalism within the framework of the currently available professional development of practicing physicians, many of whom are the residents’ clinical faculty. Consequently, formal training in professionalism for both residents in training and their faculty may lag behind organizational expectations, and residents therefore tend to learn about medical professionalism through interaction with patients, senior physicians, co-medicals and colleagues.

Previous studies reported that modern physicians encounter many challenges with regard to medical professionalism in their everyday practices [7-11]. Hodges BD et al. suggested that there are different ways of thinking about professionalism that can lead towards a multi-dimensional, multi-paradigmatic approach to assessing professionalism [12]. In 2005, we conducted a study using a questionnaire developed by Barry and colleagues [13] with challenging scenarios to professionalism, considered to be one of the more commonly used tools based on practical scenarios to measure professionalism, and found that many physicians at various levels of training in Japan also encounter challenges when responding to these common scenarios. Many Japanese physicians also report that they are concerned with their inadequate training in this area [11]. However in the years surrounding our 2005 survey, Japan introduced a national mandatory residency program and we noted that teaching hospitals improved their educational environments, including appropriate behaviors related to professionalism [14], but little additional information is available on the explicit evaluation of medical professionalism among Japanese physicians.

In this context, it is important to investigate the current state of professionalism in the Japanese medical education environment, and to examine if the level of medical professionalism among medical trainees has improved in Japan between 2005 and 2013.

## Methods

### Design, setting and participants

We conducted a survey of resident physicians at multiple teaching hospitals in Japan, using the Barry Questionnaire [13]. Permission has been granted for the use of this instrument [2]. The questionnaire instrument contains six challenging cases to professionalism: acceptance of gifts; conflict of interest; confidentiality; physician impairment; sexual harassment; and honesty in documentation. Each scenario was followed by 4 or 5 possible responses. After completing all six scenarios, participants were asked about their demographics and information on their hospitals.

We used existing nationwide general medicine in-training examination (GM-ITE) for conducting the questionnaire in these hospitals [15]. This 100-item (total score of 100) case-based examination was conducted by the Japan Organization of Advancing Medical Education Program (JAMEP, a non-profit organization) and was held for three hours once in year 2013 in March, the last month of Japanese academic year for Postgraduate Year (PGY) 1 and 2 resident physicians. The invitation to participate in the study was announced on the JAMEP website, a monthly medical journal advertisement (*Resident Note*, Yodosha, Tokyo, Japan) and was sent to program directors of all teaching hospitals (n = 1,015) in Japan certified by Ministry of Health, Labour and Welfare, including 114 university hospitals and 901 community teaching hospitals.

The program director at each hospital was required to assemble residents in a room at each hospital at a scheduled time, and provide the GM-ITE for their residents. Each program director then collected the completed examination answer sheets, and returned them to JAMEP using an envelope we had provided. All surveys were conducted in March, 2013.

### Questionnaire

The Barry Questionnaire was developed and validated in a study conducted in Colorado, USA by Barry et al. [13]. They performed the following steps to develop and evaluate the instrument: the scenario review was conducted by a panel of people with experience in medical ethics, clinical practice, or laws; the consensus on the “best response” and the “second best response” for each scenario was derived. We present each scenario of the Barry Questionnaire with the best response and the second best response in the Appendix. In our previous pilot study, we developed the Japanese version by translating texts and responses of the instrument into the Japanese language [11]. Also in the previous study, content validity of this Japanese version was confirmed by an independent panel comprised of physicians responsible for educational programs in participating hospitals, based on the contents of professionalism guideline of the Japanese Medical Association [16].

### Analysis

We analyzed the frequency of providing either the best or the second best answers to each scenario as a main outcome measure, and compared those frequencies between PGY-1 and PGY-2 resident physicians, between men and women physicians, and also between residents of university hospitals and those of community teaching hospitals. We also compared the main outcome measures of the current study to those of our previous pilot study [11] and also to US residents from the original study by Barry and colleagues [13]. The Chi-Square Test was used to analyze data for comparing proportions. Student’s t-test was used to analyze data for comparing mean scores. All statistical analysis was performed using IBM SPSS Statistics 21.0 (IBM Japan, Tokyo, Japan), and two-tailed p-value of less than 0.05 was considered as statistically significant.

### Ethics statement

All participants, whose native language is Japanese, read and signed informed consent before the survey. Participants were assured of confidentiality and anonymity. They had the option to decline participation or terminate participation during the course of the study. The study was conducted in compliance with the Helsinki Declaration and was approved by the Institutional Review Board of Mito Kyodo General Hospital, Mito city, Ibaraki, Japan (reference No. 11–18).

## Results

A total of 431 PGY-1 and 618 PGY-2 resident physicians-in-training (290 women, 28%) participated and completed the survey. Of the PGY-2 physicians, 159 (26%) were women and 459 (74%) were men. Of the PGY-1 physicians, 131 (30%) were women and 300 (70%) were men. Among all participants, the mean score of the exam was 58.1 (standard deviation [SD], 7.5). There were 114 teaching hospitals. Nine hospitals (7.9%) were university hospitals, while 105 hospitals (95.6%) were community hospitals.

Overall, residents performed better for five scenarios (gifts and honesty) but not for scenario of harassment compared to the results of the survey conducted in 2005. The greater proportions of the best or 2nd best responses were identified in issues about gifts (n = 853, 81.3%) and honesty (n = 991, 94.5%). The proportion of the best or 2nd best responses to the harassment was lower (n = 336, 32.0%) in the 2013 survey than that of the 2005 survey.

Table 1 compares the frequencies that the participants provided the best or 2nd best responses between PGY-1 and PGY-2 residents. The frequency of either the best or 2nd best responses to the gifts (p = 0.002) and harassment (p = 0.031) scenarios were significantly greater in PGY-2 residents than in PGY-1.

**Table 1:** **The frequency of the best or 2nd best responses for each scenario: PGY1 and PGY2**

	PGY1 (N = 431)		PGY2 (N = 618)		p-Value
	n	%	n	%	
Gifts	331	76.8	522	84.5	0.002
Conflict of Interest	317	73.5	479	77.5	0.14
Confidentiality	353	81.9	492	79.6	0.36
Impairment	409	94.9	582	94.2	0.62
Harassment	122	28.3	214	34.6	0.031
Honesty	403	93.5	588	95.1	0.25

The results of comparing the frequencies of the best or 2nd best responses between residents of university hospitals and community hospitals are presented in Table 2. The frequency of either the best or 2nd best responses to the gifts (p = 0.006) and honesty (p = 0.001) scenarios were significantly greater in residents of community hospitals than of university hospitals.

**Table 2:** **The frequency of the best or 2nd best responses for each scenario: university hospitals and community hospitals**

	University (N = 186)		Community (N = 855)		p-Value
	n	%	n	%	
Gifts	138	74.2	708	82.8	0.006
Conflict of Interest	135	72.6	653	76.4	0.27
Confidentiality	157	84.4	681	79.6	0.14
Impairment	172	92.5	812	95.0	0.18
Harassment	70	37.6	263	30.8	0.069
Honesty	167	89.8	818	95.7	0.001

Table 3 shows the results comparing the frequencies of the best or 2nd best responses between men and women residents. The frequency of either the best or 2nd best responses to the gifts (p = 0.005) scenario was significantly greater in men than women.

**Table 3:** **The frequency of the best or 2nd best responses for each scenario: men and women**

	Men (N = 759)		Women (N = 290)		p-Value
	n	%	n	%	
Gifts	633	83.4	220	75.9	0.005
Conflict of Interest	568	74.8	228	78.6	0.2
Confidentiality	607	80.0	238	82.1	0.44
Impairment	720	94.9	271	93.4	0.37
Harassment	230	30.3	106	36.6	0.052
Honesty	715	94.2	276	95.2	0.54

Table 4 compares the performance of residents of the current survey in 2013 with that of our previous pilot study in 2005. The frequency of either the best or 2nd best responses to the gifts and honesty scenarios were significantly greater in the current residents than those in previous study [11]. However, the frequency of either the best or 2nd best responses to the harassment scenario was significantly greater in participants in the previous study than those in the current study. Table 5 shows the frequency of each response to harassment scenario in total and by gender, since the frequency of the best or 2nd best responses to this scenario were lower than those of other scenarios. Many residents choose the B response (Report the incident to the program director as an example of sexual harassment), which was chosen more frequently in men than in women. Only two men and no women chose the response A (Do nothing, on the basis that the faculty member was simply showing his appreciation for a job well done).

**Table 4:** **The frequency of the best or 2nd best responses for each scenario: Our previous study in 2005 and this study in 2013**

	2005 (N = 60)		2013 (N = 1049)		p-Value for 2005 vs 2013
	n	%	n	%	
Gifts	33	55.0	853	81.3	<0.001
Conflict of Interest	49	81.7	796	75.9	0.31
Confidentiality	54	90.0	845	80.6	0.07
Impairment	56	93.3	991	94.5	0.71
Harassment	30	50.0	336	32.0	0.004
Honesty	39	65.0	991	94.5	<0.001

**Table 5:** **The frequency of each response to harassment scenario in total and by gender**

	Men		Women		Total	
	n	%	n	%	n	%
A	2	0.3	0	0	2	0.2
B	349	46	117	40.3	466	44.4
C (Best)	117	15.4	61	21	178	17
D	177	23.4	67	23.1	244	23.3
E (2nd best)	113	14.9	290	15.5	158	15.1

## Discussion

Based on the results of the current study, many more PGY-2 Japanese physicians were able to provide an “acceptable” response to challenges to professionalism on several issues compared to PGY-1 physicians regarding gifts and harassment issues. Residents in community teaching hospitals provided the greater number of acceptable responses to professionalism issues than those in university hospitals in terms of gifts and honesty issues. Moreover, residents in the current 2013 study responded more appropriately to professional challenges than those in our previous study in 2005, specifically gifts and honesty issues with statistical significance.

There are numerous interesting aspects to this study. First, the validation of the study by the Japan Medical Association is gratifying. It is important that medical leadership within the private, university and government sectors explicitly support the teaching and assessment of professionalism (and the other core competencies as well) at all levels. It would be extremely interesting to elicit the responses of these and other medical leaders and teachers to the professionalism scenarios of the study.

### Gifts

A previous study [17] on the relationship between Japanese physicians and the pharmaceutical industry showed that many physicians received gifts from pharmaceutical companies, indicating the close relationship between Japanese physicians and pharmaceutical industries. Recently, however, there has been active discussions concerning the relationship between clinical physicians and pharmaceutical companies in Japan. Several authors have emphasized receiving those gifts should be regarded as inappropriate relationship between physicians and pharmaceutical companies [18,19]. Many residency programs introduced faculty development education recommending against receiving those gifts. Therefore, recent residents may have learned more ethically appropriate relationships with drug companies than the earlier study group.

### Harassment

In addition, Japanese society has recently supported policies and rules against several forms of harassment, including sexual, power, and academic issues. Many industries, schools, and hospitals have introduced local committees for preventing or managing these issues. Thus, PGY-2 residents may have been introduced to more appropriate behaviors about sexual harassment. However, the proportions of acceptable response to the harassment scenario were lower than those of other issues. The response B (Report the incident to the program director as an example of sexual harassment.) was selected most frequently (44%) in the current study. Based on the case scenario (Additional file 1), if the witnessed person (the person observing the incident and answering the questionnaire) is a resident, and the “suspect” is a faculty member, many residents would answer the response B because it seems difficult to confront or approach someone in power position on this topic. There may be limitations on accurately evaluating this item: do we recognize this behavior as inappropriate and unprofessional and how do we deal with it as individuals when witness and suspect are in equal or different positions, same or different gender? Fortunately, no women and very few (2 men) chose the response A, which was more problematic because of under-recognition of the harassment behavior. It is clear that the sexual harassment policy of each Japanese teaching hospital still needs to be widely circulated and discussed, and they should provide reporting procedures and counseling for victims and witnesses.

### Gender differences

Gender differences were observed in this study. The question of gender is of great interest in all societies. The position of women within medicine occurs within historic and cultural context, and has been changing in recent years. Men performed significantly better in terms of gift scenario than women. One possible explanation would be that women may tend to avoid the interpersonal conflicts by accepting gifts from someone even recognize that it is inappropriate. The other potential explanation may be that women are self-confident about the independence between their financial relationship with pharmaceutical companies and their professional decision-making with regard to medical care. A study on Japanese medical students suggested that women were different from men in terms of greater feelings of empathy in women students [20]. Regarding the sexual harassment scenario, there was no gender difference of the responses in the current study, although this scenario might reflect more greatly to gender difference. In Japan, men and women may recognize this issue relatively equally. There were no data about whether the proportion of women in the workforce may be related to attitudes and further study is needed for exploring this point.

### Professionalism curricula

On the different level, professionalism curricula itself is undergoing evolution in many countries. Like all educational change, the process is slow or even arduous, and subject to many factors; not the least of which are the many competing priorities for faculty time and attention. There is the paucity of data on “what works” in professionalism education and assessment. Since both “hard” outcomes and process data are sparse, it is especially challenging to interpret the current study’s results as a function of the specific curricula at participating medical schools and residencies. Having said that, it is reassuring to note the very helpful anecdotal references on core competency training on the ABIM website [21-23].

### Hidden curricula

Of course, while many physicians, both in training and “after” training, perceive that professionalism education is lacking, all professionals participate in what has been termed the “hidden curriculum” [24,25]. Most professionals know what the “rules” are or ought to be, the hidden curriculum of hallways, call rooms, and elevators teach the evolving physician how to deal with the “reality,” practically and safely if not purely “ethically.” The hidden curriculum of expedience and survival cannot be ignored or, by itself, dismissed or criticized. Only when “it” is acknowledged, and subjected to a candid and “safe” discussion, including explicit inquiry into the challenges faced especially by physicians in early training, can we hope to support professional behavior which is more consistent with formal guidelines for professional and ethical behavior [26]. In Japan, medical professionalism had been considered as unwritten contract with society or a matter of course based on “noblesse oblige” [3,4]. However, as an old fashioned code of personal conduct has lessened in recent years, it is getting more important not to leave professionalism to each individual.

### Community versus university hospitals

In terms of issues about gifts and honesty, many more residents in community teaching hospitals provided the greater number of acceptable responses than those in university hospitals. After introduction national mandatory residency program by Japanese Ministry of Health, Labour and Welfare, community teaching hospitals improved their educational environments which were proved to be better than those of university hospitals [14]. The better hospital educational environment included appropriate behaviors related to professionalism.

### Professional development

Lastly, the results here strongly support the development over time of professional ethics, specifically from PGY-1 to PGY-2. It is clear that the evolution of all aspects of professional “core competencies” has developmental aspects. Clearly, there are no discrete “stages” in the evolution of professional beliefs or behavior, but we do change with experience, exposure, repeated daily challenge, individual introspection and collegial consultation and discussion, both formal and informal. An important opportunity for educators and trainees alike is to view daily practice as a learning “laboratory.” Similar to the utility of problem-based learning approaches elsewhere in medical education, the everyday challenges of hierarchy, temptation of all kinds, the myriad of ethical and communication dilemmas inherent in an imprecise, uncertain, and often high stakes profession all provide endless opportunities for explicit inquiry, exploration, reflection and discussion. In fact, it is an authors’ belief that the process of honest and ongoing professionalism dialogue is more important than the articulation of specific rules. In fact, it is only through such dialogue that we have any hope of “playing by the rules”.

### Limitations

There are several limitations in our study. The first limitation was low participant rate. Only 1,049 out of 15,800 residents (about 7%) participated in our survey. However, our sample size (over 1,000) was relatively large given that there were about 15,800 PGY-1 and PGY-2 residents in Japan. The second limitation may be different views by physicians in different cultures. Some scenarios may not be encountered commonly and several responses could be viewed as acceptable behaviors in Japanese practice settings. Third, given that only 1,049 of about 15,800 residents throughout Japan participated in this study, our findings may not be generalizable to all residents in Japan. Program directors are responsible for decision to participate in this exam. However, no residents who received the GM-ITE denied the participation for this survey and there was little space for bias toward the participation of greatly professional residents. Fourth, the current study differs in size and design from the 2005 study, which was small in sample size and used a convenient sample with data collection involved not at the same time as an exam. A framing effect might have been operated in the current study to lead respondent to more socially desirable responses than at other times. Thus, the results might not have reflected the true change. Fifth, there has been no notable change in the professionalism curricula over the last 8 years in the formal residency outcomes project initiated by the Japanese Ministry of Health after the introduction of national mandatory residency programs. However, especially in community teaching hospitals, clinician educators have been encouraged to take leading roles for resident education partially motivated for recruiting the greater number of residents. This behavioral trend might have been a cause for better responses to some issues, including gifts and honesty scenarios. Regarding gifts scenario, there might not have been few practical changes (76% vs 83%) despite its statistical significance.

## Conclusions

Our results showed that residents in year 2013 were more likely to provide acceptable responses than those in year 2005, specifically gifts and honesty issues with statistical significance, suggesting significant improvement of professional behaviors in Japanese residents for recent several years. In addition, there was a learning curve effects related to professionalism during first two years of residency in Japan. Professionalism education in university hospitals should be evaluated to improve educational environment for their residents.

## Additional file

Additional file 1:
**Appendix: Barry Professionalism Questionnaire.**
